# Outcomes are what matter: Competency-based medical education gets us to our goal

**DOI:** 10.15694/mep.2018.0000085.1

**Published:** 2018-04-12

**Authors:** Shelley Ross, Karen E. Hauer, Elaine van Melle

**Affiliations:** 1 University of Alberta; 2 University of California San Francisco; 3 Royal College of Physicians and Surgeons of Canada

**Keywords:** competency based medical education

## Abstract

This article was migrated. The article was marked as recommended.

Health professions education is undergoing a major paradigm shift to competency-based medical education. When shifts in thinking are profound and result in transformations of existing paradigms, there is often an accompanying criticism. While competency-based medical education is an evidence guided change in approach to curriculum and assessment, it is not immune to critique and concerns. Some criticisms are valid, and must be addressed as competency-based medical education is implemented; other concerns raised about competency-based medical education highlight the importance of clarity in language and purpose when discussing new paradigms. In this commentary, we aim to offer a balanced view of competency-based medical education by presenting an overview of the origins and conceptual assumptions of competency-based medical education and acknowledging valid criticisms of the approach.

## Commentary

Competency-based medical education (CBME) is now entrenched in medical education across Canada, the United States, and parts of Europe and the United Kingdom. ^1^ Inevitably, this profound and widespread shift in educational approach is accompanied by criticisms of CBME - including questioning the need for change, danger of reductionism, and lack of evidence for CBME. However, in light of rising concern over preventable medical errors, it is difficult to refute that existing time-based medical education models are falling behind in preparing graduates for increasingly complex systems of patient care. CBME is an evidence-guided alternative to an outdated educational approach that must be changed.

Healthcare systems and patient care needs are changing dramatically. The goal of CBME is to create physicians who are able to meet those needs, and improve patient care. In order to provide optimal care, physicians must be increasingly competent in skills such as communication, professionalism, teamwork, and patient-centred care; skills that go well beyond the traditional emphasis on attaining and applying knowledge. ^2^ By focusing on learner outcomes and the competencies required for practice, CBME is well positioned to prepare physicians for this brave new world of healthcare.

The CBME framework requires fundamentally changing our approach to medical education ( Table 1). The central premise of CBME is that the competencies required for optimal patient care outcomes are clearly defined and guide the design of all curricular elements. CBME therefore requires substantial redefinition of assessment practices as well as faculty and learner roles, responsibilities, and relationships. ^3^Indeed, in CBME, each learner is viewed as having an individual developmental path with unique strengths and areas for improvement. CBME is therefore not a tweak to the system; it is a holistic, transformative change in how we prepare physicians for practice. CBME focuses on outcomes: “What can our trainees **do**?”, rather than “Did our trainees complete the curriculum?” The focus on the outcome of demonstrated learner competence in workplace settings achieves the critical connection between CBME and patient care needs.

**Table 1.** Competency-based medical education is a fundamentally different approach than traditional medical education

Competency-based medical education	Time-based medical education
Emphasis is on what the trainee **does**	Emphasis is on what the trainee **knows**
CBME is an evidence-guided intervention, drawing from multiple theories and approaches within and beyond medical education (e.g., mastery learning, programmatic assessment, assessment *for* learning) to achieve pre-defined learning outcomes	Traditional medical education addresses what is taught, including when and for how much time
Relationship between the trainee and teachers is one of coaching towards competence	Relationship between trainee and teachers is primarily unidirectional, with teachers imparting knowledge/skills (teaching) or judging (assessing) the trainee
Learning experiences are guided by individual progress towards competence	Learning experiences are defined by fixed curricula with specific allotments of time
Reflection on learning, self-monitoring, and self-regulated learning are explicitly expected, and are supported by the faculty coach	Learners are recipients of teaching and have limited choice in what they learn, when they learn it, or in directing their own learning
Learning is individualized to the greatest degree possible, allowing learners to focus on enhancing learning in self-selected areas once competence is demonstrated, or adding time if needed to demonstrate competence	Learning is assumed to be “one size fits all”, with limited flexibility to meet individual needs
Reflection and self-monitoring carry on into continuing professional development	Continuing professional development is not seen as a continuation of the learning during training

The transformational aspect of CBME poses challenges for many. For example, critics assert that CBME is too reductionistic, and that various competency frameworks like CanMEDS and the ACGME competencies parse “being a good doctor” into individual checklists that discount the whole. This valid criticism represents a risk in CBME implementation that must be monitored, lest CBME devolve into an ineffectual list of tick boxes. CBME is about creating clarity in outcomes, but not while losing sight of the learner as a developing healthcare professional. CBME requires looking at medical education similarly to how we approach caring for a complex patient with multiple co-morbidities. While we need to look at each element and treat them individually, the whole person remains a priority at every stage of management.

Ironically the word “competence” itself is a target of criticism: “we should train for excellence, not mere competence or minimally acceptable performance”. Rather than lowering the bar, the intent of CBME is to promote “ *cumulative learning along a continuum of increasing medical sophistication.*” ^3,p.19^ As an evidence-guided practice, CBME draws substantially from mastery learning and also incorporates existing, evidence-based educational theories and approaches from across the educational continuum within and beyond medical education. ^4^ Furthermore, as is consistent with advanced studies on promoting optimal learning, CBME stresses direct observation - educators must watch trainees with patients and colleagues to ensure demonstration of competence. The faculty role evolves from transmitter of information to observer, coach, and guide. Again, as is consistent with our evolving understanding of building capacity for life-long learning, trainees are challenged to take responsibility to direct and enhance their own learning. ^5^ Ultimately, competence at graduation is not the end point, it is the starting point for a lifetime of learning which builds on that attained and demonstrated competence. CBME continues into practice, as a fundamental part of continuously learning and improving as a physician.

“Where is the evidence for CBME?” - critics ask with heightening urgency. This relevant question reflects the reality that most CBME work to date has focused on applying core theories of learning into practice, rather than creating evidence of validity. Early research is beginning to show that greater amounts of feedback and direct observation, both inherent aspects of CBME, improve the capacity of the program to respond to individual learning needs, including the early detection of residents in difficulty. Going forward however, it is important to acknowledge the need to accumulate evidence regarding the impact of CBME. It is naïve however, to assume that one study can definitively prove the value of CBME. CBME is a complex intervention with multiple components, and creating evidence that CBME ‘works’ will require systematic accumulation of data - what works for whom under which circumstances and why. ^6^ The challenge is to focus on understanding the implementation chain - how the various CBME inputs and activities contribute to intended and unintended outcomes. This chain mimics clinical research, where accumulation of evidence progresses from theory and observation to small, short term single institution interventions to larger rigorous multi-institutional studies, over time supporting evidence-informed guidelines and consensus.

Not all educators are convinced that CBME is needed, or worth pursuing. To some, CBME is seen as trendy or simply an intellectual exercise, similar to other past educational innovations (e.g., portfolios, problem-based learning), where a good concept did not always live up to its promise. To others, CBME appears to be yet another “make-work project”, similar to adding learning objectives to curricula- an arduous task that generated a lot of work, but produced little real change.

Unlike these targeted innovations, however, we have argued that CBME calls for transformative curricular change. We have also identified an urgent need to build the necessary base of evidence in order to fully understand the impact of CBME. For although CBME embodies significant advances in educational practice, we must remain aware that our focus on competencies is not about ticking off checklists but about creating physicians who are able to meet rapidly evolving societal needs. ^7^ Accordingly, as a group involved in frontline education, program leadership, and research, we welcome ongoing dialogue.

## Notes On Contributors

Shelley Ross, PhD is an Associate Professor at the University of Alberta.

Karen E. Hauer, MD, PhD is a Professor and Associate Dean, Competency Assessment and Professional Standards at the University of California, San Francisco.

Elaine van Melle, PhD is a Senior Education Scientist, Royal College of Physicians and Surgeons of Canada.

The International Competency Based Medical Education Collaborators are a group of health professions education experts who examine conceptual issues and current debates in CBME. A list of the ICBME Collaborators can be found at http://www.royalcollege.ca/rcsite/educational-initiatives/icbme/icbme-collaborators-e

## References

[1] Carraccio CL Englander R . From Flexner to competencies: Reflections on a decade and the journey ahead. Acad Med. 2013;88:1067–1073. 10.1097/ACM.0b013e318299396f 23807096

[2] Frenk J Chen L Bhutta ZA Health professionals for a new century: Transforming education to strengthen health systems in an interdependent world. Lancet. 2010;376:1923–1958. 21112623 10.1016/S0140-6736(10)61854-5

[3] McGaghie WC Miller GE Sajid AW Telder TV . Competency-based Curriculum Development in Medical Education: An Introduction. Public Health Papers No. 68. Geneva, Switzerland: World Health Organization;1978. 664734

[4] Hattie J Yates G . Visible Learning and the Science of How We Learn. New York, NY: Routledge; 2014.

[5] Schultz K Griffiths J . Implementing Competency-Based Medical Education in a Postgraduate Family Medicine Residency Training Program: A Stepwise Approach, Facilitating Factors, and Processes or Steps That Would Have Been Helpful. Acad Med. 2016 May 1;91(5):685–9. 10.1097/ACM.0000000000001066 26717504

[6] Pawson R Greenhalgh T Harvey G Walshe K . Realist review - a new method of systematic review designed for complex policy interventions. J Health Serv Res Policy. 2005.10:S21–34. 10.1258/1355819054308530 16053581

[7] Halman M Baker L Ng S . Using critical consciousness to inform health professions education: A literature review. Perspet Med Educ. 2017.6:12–20. 10.1007/s40037-016-0324-y PMC5285284 28050879

