# Supplementary material for: Outcomes are what matter: Competency-based medical education gets us to our goal
Source: MedEdPublish (2016). 2018 Apr 12;7:85. [Version 1] doi: 10.15694/mep.2018.0000085.1 (PMC10711948; doi:10.15694/mep.2018.0000085.1)
Supplement: Peer review history

# Reviewer report: Reviewer response for version 1

**DOI:** 10.21956/mep.19557.r28994

**Published:** 2018-05-21

**Authors:** Sateesh Babu Arja

**Affiliations:** 1 Avalon University School of Medicine

This review has been migrated. The reviewer awarded 4 stars out of 5 It is a very well written paper and it is an important topic these days in medical education. Even in the ICME-Ottawa conference 2018 in Abu Dhabi, there was a great deal of discussion about competency-based medical education. The more emphasis was given for the competencies, skills, communication, and professionalism. I also heard the quote "Medical graduates are expected to be fit to practice rather than to be fit to pass only assessments or examinations". The other comment is knowledge is cheap and it available everywhere on the internet.Lot of medical schools is shifting towards the competency-based medical education. But we need to question ourselves as educationalists, what went wrong with current medical education or curriculums as mentioned by professor Puthiaparampil? Are we not preparing the competent enough physicians? I would like to see more of these details. I have also seen in some other conferences regarding artificial intelligence and/or robots taking over examination skills and/or diagnosis and left over is only communication and professionalism to the medical profession and physicians. If this medical profession left only with communication and professionalism, is current medical education preparing the graduates competent enough in communication skills and professionalism? Or do us really need competency-based medical education. I would like to see all these to be answered before us judging competency-based medical education and current medical education.I would like to add one more comment that when we are making a change, we need to acknowledge the merits in the current system. Even though there is less importance for didactic lectures and imparting knowledge in the current days, I believe that lectures provide the scaffold that learners need to learn.

Reviewer Expertise:

NA

No decision status is available

# Reviewer report: Reviewer response for version 1

**DOI:** 10.21956/mep.19557.r28996

**Published:** 2018-05-06

**Authors:** Michael SH Wan

**Affiliations:** 1 University of Notre Dame

This review has been migrated. The reviewer awarded 4 stars out of 5 A well written review of the CBME with good comparison of the 'classical' vs 'new competency' approach to teaching. The 'does' level is difficult to assess objectively compared to the 'classical' testing of the 'knows'. The trend for patient-centered care, clinical competency and EPAs with the rapid evolution of programmatic assessment will need good evidence based support. The limited time for the medical program with the ever expanding knowledge required for the support to become a all-rounded physician is always a challenge to the curriculum developer and assessors.

Reviewer Expertise:

NA

No decision status is available

# Reviewer report: Reviewer response for version 1

**DOI:** 10.21956/mep.19557.r28998

**Published:** 2018-04-25

**Authors:** Thomas Puthiaparampil

**Affiliations:** 1 UNIVERSITI MALAYSIA SARAWAK

This review has been migrated. The reviewer awarded 4 stars out of 5 Well written article. Surely all medical schools aim to produce efficient medical doctors. In some parts of the world it is called Outcome-Based Medical Education. I believe CBME and OBME refer to the same ideal. I would have liked to see also a detailed description of what is wrong with the current system (I am not sure what it is called). Has the current system failed so badly? Have the current doctors not performing satisfactorily? I believe all medical schools test the candidate multiple times to assess their competency in clerking real patients to reach a diagnosis, produce a plan of investigation and treatment. I can see the downside of time-based curriculum (fixing the number of years the candidate can take), which will compel the candidate either to pass the examination or leave the course. It will be excellent, if personal mentoring is given to all candidates. But how far it is practicable? I do not believe that a candidate who graduates from a medical school is already competent to practice without supervision. The 2-year houseman ship practiced in Malaysia is the time the new graduates really get the opportunity to put into practice what he learned in the medical school. One cannot ignore the strong foundation in knowledge and clinical skills which enable the doctor to perform his job efficiently. Communication skills, professionalism, team work etc. will surely follow during the 2 year houseman ship period.

Reviewer Expertise:

NA

No decision status is available

# Reviewer report: Reviewer response for version 1

**DOI:** 10.21956/mep.19557.r28993

**Published:** 2018-04-15

**Authors:** Alan Jaap

**Affiliations:** 1 University of Edinburgh Medical School

This review has been migrated. The reviewer awarded 4 stars out of 5 A really thought-provoking commentary on the current state of play for CBME. It would have been useful to expand the discussion to consider outcomes in more detail: while few would disagree that individual competencies are important, some appear too nebulous to assess unless packaged in a meaningful outcome - like an authentic clinical task. This is why the concept of entrustable clinical activities is so intuitive and appealing.A second practical aspect of CBME that warrants further consideration is the ‘time versus competency’ dilemma - i.e. how do you truly ensure competency-based progression within rigidly time-defined undergraduate courses or postgraduate training programmes? It is possible to envisage faster-track and slower-track streams with more or less elective components for those who achieve desired competencies earlier or later than average, although this would pose significant administrative challenges.Like the authors, I look forward to more publications addressing the practicalities of CBME.

Reviewer Expertise:

NA

No decision status is available

# Reviewer report: Reviewer response for version 1

**DOI:** 10.21956/mep.19557.r28997

**Published:** 2018-04-13

**Authors:** Davinder Sandhu

**Affiliations:** 1 Royal College of Surgeons in Ireland, Medical University of Bahrain, POBox 15503, Adliya, Bahrain.

This review has been migrated. The reviewer awarded 5 stars out of 5 An important paper. I enjoyed reading this paper as well as Prof Cookson's review. It is difficult to argue against a competency based curriculum. Nobody can deny that what a doctor can do is more important and better for patients rather than how long they have spent in gaining that knowledge. Best to avoid one camp or the other as all these areas are intimately related. Performance does have a direct relationship with knowledge. The judgement and higher order critical thinking is essential. A key function for a health professional is after all problem solving. I would agree that greater amount of feedback and direct observation are the way forward. The authors may wish to read the work of Profs Hilliard Jason and Jane Westberg, as in some ways the essence of their paper is also about adaptive education and supporting individual needs of the students. Practical examples of how the CBME was implemented would have been useful.

Reviewer Expertise:

NA

No decision status is available

# Reviewer report: Reviewer response for version 1

**DOI:** 10.21956/mep.19557.r28995

**Published:** 2018-04-12

**Authors:** John Cookson

**Affiliations:** 1 University of Worcester

This review has been migrated. The reviewer awarded 4 stars out of 5 I found this a well written and well argued case for competency-based medical education. Nevertheless I feel that ‘it’s a bit more complicated than that’. No one would deny that we need competent doctors. We can also recognise that lists of ‘competencies’ to be ‘ticked off’ are antithetical to real education. We must be aware that doctors who have ‘achieved all the competencies’ are not necessarily competent. Some of the most important attributes, such as self-awareness, response to feedback and so on, are the ones that are most difficult to define and therefore to assess. Expert judgement based on observation is good, multiple expert judgements even better but without a clear metric may be subject to error and difficult to defend in progress decisions. Progressive development towards a goal is fine but sooner or later someone, somehow, has to make a dichotomous decision; pass or fail.

Reviewer Expertise:

NA

No decision status is available
